# Equity and efficiency of health resource allocation in the Chengdu–Chongqing Economic Circle of China

**DOI:** 10.3389/fpubh.2024.1369568

**Published:** 2024-08-27

**Authors:** Tianqi Wang, Ting Zhou, Leming Zhou, Yunfei He, Jian Wang, Yonghong Wang, Li Huang

**Affiliations:** ^1^School of Public Health, Chongqing Medical University, Chongqing, China; ^2^College of Computer Science and Technology, Chongqing University of Posts and Telecommunications, Chongqing, China; ^3^Department of Personnel, The First Affiliated Hospital of Chongqing Medical University, Chongqing, China; ^4^Department of Pathophysiology, Chongqing Medical University, Chongqing, China; ^5^Department of Clinical Laboratory, Chongqing Qianjiang Central Hospital, Chongqing, China

**Keywords:** Chengdu–Chongqing Economic Circle, health resource allocation, equity, three stage DEA, Malmquist productivity index

## Abstract

**Objective:**

This study aimed to evaluate the fairness and efficiency of health resource allocation (HRAE) in Chengdu-Chongqing Economic Circle after the new healthcare reform. This study also aimed to identify existing problems, providing empirical evidence for the government to formulate regional health plans scientifically and reasonably.

**Methods:**

The fairness of health resource allocation was analyzed using the Gini coefficient, Theil index, and agglomeration degree from population and geographical area perspectives. The three-stage data envelopment analysis and the Malmquist productivity index were used to analyze HRAE from static and dynamic perspectives.

**Results:**

The Gini coefficient for population allocation in Chengdu-Chongqing Economic Circle was 0.066–0.283, and the Gini coefficient for geographical area allocation was 0.297–0.469. The contribution rate within a region was greater than that between regions, and health resources were mainly concentrated in economically developed core areas. The overall fairness of Chengdu Economic Circle was relatively better than that of Chongqing Economic Circle. Moreover, the adjusted mean technical efficiency was 0.806, indicating room for HRAE improvement in Chengdu-Chongqing Economic Circle. Stochastic Frontier Analysis found that different environmental variables have varying degrees of impact on HRAE. The adjusted mean total factor productivity change (Tfpch) was 1.027, indicating an overall upward trend in HRAE since the new healthcare reform. However, scale efficiency change (Sech) (0.997) limited the improvement of Tfpch.

**Conclusion:**

The fairness of health resources allocated by population was better than that allocated by geographical area. The unfairness of health resources mainly stemmed from intra-regional differences, with considerable health resources concentrated in core areas. Over the past 13 years, HRAE has improved but exhibited spatial heterogeneity and Sech-hindered productivity improvement. The study recommends strengthening regional cooperation and sharing to promote the integrated and high-quality development of the health and well-being in Chengdu–Chongqing Economic Circle.

## 1 Introduction

The rapid growth of the global economy and society has brought about tremendous challenges. These include an aging population, the transformation of diseases, and an increase in chronic disease patients (1). China initiated a critical and demanding healthcare reform in 2009 to tackle these challenges and fulfill the public’s evolving health needs (2, 3). This reform recognizes the imbalanced development of regional medical and health undertakings and unreasonable resource allocation in the medical and health field (4). It proposes to strengthen regional health planning and encourage co-construction and sharing, pointing out the development direction for optimizing the allocation of regional health resources.

The Chengdu-Chongqing Economic Circle is located at the intersection of the Belt and Road and the Yangtze River Economic Belt. Since China’s 11th Five-Year Plan (5), the development of the Chengdu-Chongqing Economic Circle has been at the core of the national development strategy, making a significant contribution to the country’s overall progress. The Chengdu-Chongqing Economic Circle Development Plan Outline (Planning Outline), released in 2021, particularly emphasizes the optimization of medical resource allocation. The Chengdu-Chongqing Economic Circle is not only the most densely populated area in Western China (6), but also faces the challenge of an aging population. The demand for medical and health services among residents is rapidly increasing in a diversified and multi-level manner. With support from national policies, it has achieved specific results in allocating health resources, which continue to grow. For example, it has established an alliance for the development of Traditional Chinese Medicine, accelerated the construction of national regional medical center projects, and built a cancer prevention and control community. However, the vast geographical area and complex mountainous terrain have to some extent limited the effective flow of health resources (7). Faced with these challenges, the Chengdu-Chongqing Economic Circle still has many issues in the balance of supply and demand of health resources, including the imbalance of resource allocation and insufficient allocation efficiency. Therefore, the rational allocation of limited medical and health resources has become particularly critical in promoting the development of the Chengdu-Chongqing Economic Circle. An efficient health resource allocation system not only forms the cornerstone of regional development but also provides a solid medical guarantee for attracting and retaining key personnel. Improving the equity, accessibility, and utilization rate of health resources (8) is the core goal pursued by health decision-makers and health systems (9, 10). This is conducive to achieving a balance of supply and demand for medical services, jointly building and sharing basic public health services, meeting people’s needs for medical resources, improving public health literacy, promoting the comprehensive development of the Chengdu-Chongqing Economic Circle, and advancing the construction of a healthy China (11). Through scientific planning and precise investment, the allocation of medical and health resources in the Chengdu-Chongqing Economic Circle will be further improved, providing strong support for the region’s sustained prosperity and the health and well-being of the people.

Previous research on health resource allocation has mainly focused on the national level or particular provinces or cities, and the research content is limited to a single aspect of fairness or efficiency. Liu et al. (12) analyzed the trends and equity of health resource allocation in primary-level medical and health institutions in China during the 13th Five-Year Plan period, finding that equity in health resource allocation in primary-level medical institutions in the eastern, central, and western regions of China has been continuously improving, but there are still differences. Wang et al. (13) used the Lorenz curve, Gini coefficient, and Theil index to evaluate the equity of health resource allocation in China by population and geography in 2019, discovering that the equity of health resources allocated by geographical area was much lower than that allocated by population, and that internal inequity within various regions is the main factor affecting the equity of health resource allocation in China. Fan et al. (14) against the backdrop of the construction of a tiered diagnostic and treatment system, evaluated the efficiency of health service resource allocation in Shandong Province, using the DEA-TOPSIS method for static analysis of health service resource allocation efficiency, and the DEA-Malmquist model for dynamic analysis. From 2012 to 2022, the average Malmquist Index in Shandong Province was 0.970, the average technical efficiency change index was 1.012, and the average technological progress index was 0.958, indicating that the decline in the Malmquist Index was mainly influenced by the technological progress index.

Only a few existing studies take regions as research subjects and combine equity and efficiency for comprehensive analysis. For instance, Zang et al. (15) conducted a study on the equity and efficiency of health resource allocation in the Yangtze River Delta region, using the Gini coefficient and Theil index to evaluate the equity of health resource allocation and a three-stage DEA model to assess efficiency. They found that the equity of health resource allocation by population in the Yangtze River Delta was better than by geography, with Shanghai’s geographical allocation being in an unfair state. The overall efficiency of health resource allocation in the region is relatively high, but there are inter-regional differences. Wen et al. (16) analyzed the equity and efficiency of health resource allocation among the city clusters in the Guangdong-Hong Kong-Macao Greater Bay Area and found that from 2010 to 2020, the level of inequity in health resource allocation in the city clusters of the Greater Bay Area continued to improve, although there were regional differences in allocation efficiency, with technological regression being the main reason for the decline in total factor productivity. Zhou et al. (17) used the entropy weight TOPSIS method and the rank-sum ratio method to conduct a comprehensive evaluation of health resource allocation in the Chengdu-Chongqing Economic Circle. They discovered that there were significant regional differences in health resource allocation within the circle, with the Sichuan area showing a more balanced allocation and the Chongqing area showing a more polarized allocation. However, the study did not conduct an in-depth analysis of the equity and efficiency of health resource allocation, and the robustness of the research findings, as well as the causes of the health resource allocation issues, require further exploration.

This study takes the Chengdu–Chongqing Economic Circle as the research object and comprehensively analyzes the fairness and efficiency of health resource allocation in Chengdu–Chongqing Economic Circle from 2009 to 2021. A series of research methods such as Gini coefficient, Theil index, and agglomeration degree are used to evaluate the fairness of health resource allocation from the perspectives of population and geography. This study also employs the three-stage data envelopment analysis (three-stage DEA) model and Malmquist productivity index (MPI) to evaluate the HRAE from static and dynamic perspectives. Additionally, the factors affecting HRAE are analyzed. Our research aims to provide scientific reference for promoting high-quality and balanced development of medical services in Chengdu–Chongqing Economic Circle.

## 2 Methods

### 2.1 Data sources and regional distribution

This study utilized panel data from 44 districts in Chengdu-Chongqing Economic Circle from 2009 to 2021 for empirical analysis. The data originates from the healthcare reform in 2009 and continues until the beginning year of the 14th Five-Year Plan. This duration was a critical period for promoting the construction of a healthy China and advancing medical and health system reform, including the Chengdu-Chongqing Economic Circle in the National Five-Year Plan, underscoring the importance of promoting coordinated development of Chengdu-Chongqing Economic Circle at the national level. The data sources for this study include the Sichuan Health Statistical Yearbook, Sichuan Statistical Yearbook, Chongqing Health Statistical Yearbook, and Chongqing Statistical Yearbook, and selected statistical yearbooks from various districts and counties in Chongqing.

**Figure 1:**
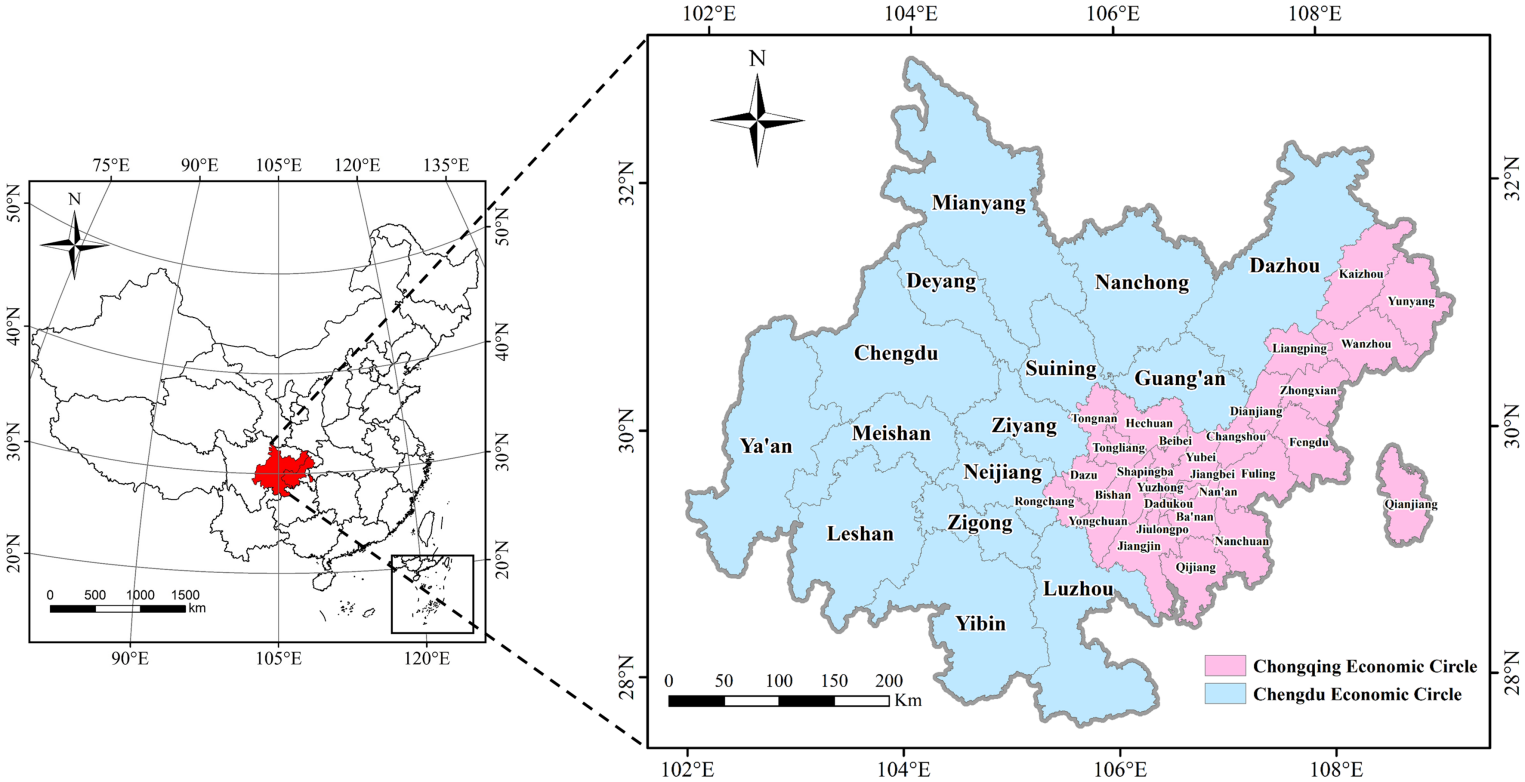
Regional distribution range of Chengdu–Chongqing Economic Circle.

This article referred to the distribution range of Chengdu-Chongqing Economic Circle proposed in the Planning Outline. And taking into account the availability of indicator data and the accuracy of research results, the study area is divided into 2 regions and 44 districts. One was that Chengdu Economic Circle included 15 prefecture-level cities such as Chengdu, Zigong, Mianyang, Suining, Luzhou, Deyang, Nanchong, Meishan, Neijiang, Leshan, Guang’an, Yibin, Ya’an, Dazhou, and Ziyang. Another was Chongqing Economic Circle, which consisted of 29 districts and counties including Wanzhou, Fuling, Yuzhong, Shapingba, Jiulongpo, Dadukou, Jiangbei, Changshou, Jiangjin, Nan’an, Beibei, Yubei, Qijiang, Dazu, Qianjiang, Ba’nan, Hechuan, Yongchuan, Nanchuan, Bishan, Tongliang, Tongnan, Rongchang, Liangping, Fengdu, Zhongxian, Dianjiang, Kaizhou, and Yunyang. The districts of Ya’an, Dazhou, Mianyang, Yunyang, and Kaizhou constituted the entire region. ArcGIS 10.8 software was used to create a regional distribution map of Chengdu-Chongqing Economic Circle (Figure 1).

### 2.2 Measuring tools

#### 2.2.1 Gini coefficients

The Gini coefficient is commonly used to evaluate the fairness of health resource allocation. Internationally, the Gini coefficient is conventionally defined between 0 and 1, and different values of Gini coefficient represent varying degrees of fairness: G < 0.2 represents absolute fairness, 0.2 < G < 0.3 represents comparative fairness, 0.3 < G < 0.4 signifies relative rationality, 0.4 is the warning line for judging whether health resource allocation is fair, 0.4 < G < 0.5 indicates relative unfairness, and G > 0.5 signifies serious unfairness (18). The Gini coefficient calculation formula (Equation 1) is as follows:


(1) $$G=1-\overset{k}{\underset{i=1}{\sum }}\left(X_{i+1}-X_{i}\right)\left(Y_{i+1}+Y_{i}\right)$$

where *G* represents the Gini coefficient value, *X_i_* is the cumulative percentage of population or geographical area of the *ith* district in Chengdu-Chongqing Economic Circle, and *Yi* is the cumulative percentage of health resources (5 indicators to measure equity) of the *ith* district in Chengdu-Chongqing Economic Circle. *k* represents the total number of districts in Chengdu-Chongqing Economic Circle.

#### 2.2.2 Theil index

Compared to the Gini coefficient, one advantage of the Theil index is that it can analyze the sources of overall inequity. Like the Gini coefficient, the Theil index ranges from 0 to 1, and the lower the value, the better the equity of health resource allocation (19). The formula (Equation 2) for calculating the Theil index is as follows:


(2) $$T=\overset{k}{\underset{i=1}{\sum }}P_{i}\log\frac{P_{i}}{D_{i}}$$

where *T* signifies the Theil index value, and *P_i_* indicates the proportion of population of the *ith* district in Chengdu-Chongqing Economic Circle. *D_i_* represents the proportion of health resources in the *ith* district of the Chengdu-Chongqing Economic Circle, and *k* denotes the total number of districts in Chengdu-Chongqing Economic Circle. The Theil index (Equation 3) can be further decomposed into intra-regional (Equation 5) Theil and inter-regional Theil (Equation 4) (18, 20), with the following decomposition formula:


(3) $$T=T_{\mathrm{inter}}+T_{\mathrm{intra}}$$

(4) $$T_{\mathrm{inter}}=\overset{n}{\underset{j=1}{\sum }}P_{j}\log\frac{P_{j}}{D_{j}}$$

(5) $$T_{\mathrm{intra}}=\overset{n}{\underset{j=1}{\sum }}P_{j}T_{j}$$

where *T_inter_* signifies the differences in health resources between the two regions of the Chengdu-Chongqing Economic Circle (Chengdu Economic Circle and Chongqing Economic Circle), whereas *T_intra_* represents the differences in health resources within the two regions of the Chengdu-Chongqing Economic Circle. *P_j_* is the proportion of population in the *j*th region, and *D_j_* is the proportion of health resources in the *j*th region. *T_j_* shows the Theil index of the two regions, and *n* shows the number of regions.

#### 2.2.3 Health resource agglomeration degree

The health resource agglomeration degree (HRAD) is a new indicator proposed by scholars like Suwei Y to evaluate the equity of health resource allocation. It can comprehensively reflect the influence of population and geographical factors on the fairness of health resource allocation. The agglomeration degree of health resources refers to the proportion of health resources concentrated in a certain area that accounts for 1% of the geographical area of the upper-level region (21, 22). The calculation formula (Equation 6) for the agglomeration degree of health resources is as follows:


(6) $$HRAD_{j}=\frac{\left(\frac{HR_{j}}{HR_{k}}\right)\times 100\%}{\left(\frac{A_{j}}{A_{k}}\right)\times 100\%}=\frac{\frac{HR_{j}}{A_{j}}}{\frac{HR_{k}}{A_{k}}}$$

Population agglomeration (PAD) refers to the proportion of population aggregated in a certain area, which occupies 1% of the geographical area of the upper-level region. The calculation formula (Equation 7) for population agglomeration is as follows:


(7) $$PAD_{j}=\frac{\left(\frac{P_{j}}{P_{k}}\right)\times 100\%}{\left(\frac{A_{j}}{A_{k}}\right)\times 100\%}=\frac{P_{j}/A_{j}}{P_{k}/A_{k}}$$

where *HRAD_j_* indicates the concentration of health resources in the *j*th district, *HR_j_* signifies the number of health resources in the *j*th district, and *HR_k_* denotes the number of health resources in the upper-level region. *A_j_* represents the geographical area of the *j*th district, and *A_k_* signifies the geographical area of the upper-level region. *PAD_j_* shows the population concentration of the *j*th district, *P_j_* signifies the population number of the *j*th district, and *P_k_* shows the population number of the upper-level region.

We usually use *HRAD_j_/PAD_j_* to evaluate the fairness of population allocation, where *HRAD_j_/PAD_j_* = 1 shows absolute fairness in health resources allocated by population in the district, *HRAD_j_/PAD_j_* > 1 indicates that an excess of health resources relative to the population in the district, and *HRAD_j_/PAD_j_* < 1 signifies that the health resources in the district are relatively insufficient compared to the population. Similarly, *HRAD_j_* = 1 indicates absolute equity in the allocation of health resources in the district based on geographical area, *HRAD_j_* > 1 shows an excess of health resources allocated by geographical area in the district, and *HRAD_j_* < 1 signifies that a shortage of health resources allocated by geographical area in the district.

#### 2.2.4 Three-stage DEA model

Fried proposed the three-stage DEA model in 2002, aiming to eliminate the impact of environmental variables and random disturbances on efficiency. In this paper, the three-stage DEA model was used to analyze the HRAE of the Chengdu-Chongqing Economic Circle. The model comprised of three stages, and the calculation steps are as follows:

In the first stage, DEAP 2.1 software was employed to calculate each district’s efficiency values and slack variables in Chengdu-Chongqing Economic Circle through a BCC model with variable returns to scale. The technical efficiency (TE) can be decomposed into scale efficiency (SE) and pure technical efficiency (PTE), and TE equals the product of SE and PTE (TE = SE × PTE) (23). TE = 1 suggests that the district is in a DEA valid state, TE < 1, and SE or PTE = 1 signifies that the district is in a weak DEA efficient state, and other situations indicate that the district is in a DEA invalid state (24).

In the second stage, Frontier 4.1 software was used, with six environmental variables as independent variables and the slack values of four input indicators as dependent variables, Equation (8) for Stochastic Frontier Analysis (SFA) model can be written as:


(8) $$\begin{array}{l} S_{mk}=f\left(Z_{k};\beta_{m}\right)+\nu_{mk}+\mu_{mk}\\ k=1,2,\cdots ,44;m=1,2,3,4 \end{array}$$

In the above formula, *S_mk_* represents the slack value of the *kth* district in the *mth* input indicator, and *f* (*Z_k_*; *β_m_*) indicates the impact of environmental factors on *S_mk_*. *v_mk_* shows random perturbation, *μ_mk_* represents management inefficiency, and the sum of the two is a mixed error term. Equation (9) for calculating the adjusted investment indicators is as follows:


(9) $$\begin{array}{l} X_{mk}^{A}=X_{mk}+\left[\max\left(f\left(Z_{k};{\hat{\beta }}_{m}\right)\right)-f\left(Z_{k};{\hat{\beta }}_{m}\right)\right]\\ +\left[\max\left(v_{mk}\right)-v_{mk}\right]\\ k=1,2,\cdots ,44;m=1,2,3,4 \end{array}$$

where $X_{mk}$ and $X_{mk}^{A}$ are the input indicators before and after adjustment, respectively (25). $\left[\max\left(f\left(Z_{k};{\hat{\beta }}_{m}\right)\right)-f\left(Z_{k};{\hat{\beta }}_{m}\right)\right]$ signifies placing the 44 districts under the same external environment, and $\left[\max\left(v_{mk}\right)-v_{mk}\right]$ represents placing the 44 districts under the same random error.

In the third stage, the DEAP 2.1 software was used to calculate the adjusted efficiency values of various districts in Chengdu-Chongqing Economic Circle through the BCC model, reflecting the true situation of HRAE in Chengdu-Chongqing Economic Circle.

#### 2.2.5 Malmquist productivity index(MPI)

The MPI can dynamically evaluate the efficiency changes of decision-making units over many years. The total factor productivity change (Tfpch) can be decomposed into changes in technical efficiency (Effch) and technological progress (Techch), and Effch can be further decomposed into pure technical efficiency change (Pech) and scale efficiency change (Sech) (26). This article used DEAP 2.1 software to calculate the Tfpch value of the Chengdu-Chongqing Economic Circle in the past 13 years. The specific calculation formula (Equations 10–13) is as follows:


(10) Tfpch=Techch×Effch=Techch×Pech×Sech

(11) $$\mathrm{Tfpch}=\left(x^{t},y^{t},x^{t+1},y^{t+1}\right)={\left[\frac{E^{t}\left(x^{t+1},y^{t+1}\right)}{E^{t}\left(x^{t},y^{t}\right)}\times \frac{E^{t+1}\left(x^{t+1},y^{t+1}\right)}{E^{t+1}\left(x^{t},y^{t}\right)}\right]}^{\frac{1}{2}}$$

(12) $$\mathrm{Effch}=\frac{E^{t+1}\left(x^{t+1},y^{t+1}\right)}{E^{t}\left(x^{t},y^{t}\right)}$$

(13) $$\mathrm{Techch}={\left[\frac{E^{t}\left(x^{t+1},y^{t+1}\right)}{E^{t+1}\left(x^{t+1},y^{t+1}\right)}\times \frac{E^{t}\left(x^{t},y^{t}\right)}{E^{t+1}\left(x^{t},y^{t}\right)}\right]}^{\frac{1}{2}}$$

In the above formula, (*x^t^*, *y^t^*) and (*x*^*t* + 1^, *y*^*t* + 1^) represent the input and output indicators for the periods *t* and *t* + 1 respectively, while *E^t^* and *E*^*t* + 1^ show the distance functions for the periods *t* and *t* + 1, respectively. Each efficiency change value = 1 indicates that the efficiency remains unchanged, each efficiency change value >1 suggests an improvement in efficiency, and each efficiency change value <1 shows a decrease in efficiency (27).

### 2.3 Indicator selection

Based on the principles of representativeness, correlation, and availability selected by DEA indicators. After consulting with experts and reviewing prior research (28–30), in order to fully reflect the allocation of health resources in Chengdu-Chongqing Economic Circle, the number of medical and health institutions (MHI), actual number of beds (AB), number of practicing (assistant) physicians (PAP), and number of registered nurses (RN) were selected as input indicators. And the number of diagnoses and treatments (DT), number of surgeries performed (SP), and number of discharged patients (DP) were selected as output indicators. Considering the impact of environmental factors such as economy, finance, population, education, and society on HRAE, gross domestic product (GDP), health expenditure (HE), number of permanent residents (PR), number of primary and secondary school teachers (FTPS), urbanization rate (UR), and general public budget revenue (GPBR) were selected as environmental variables. A significant difference has been observed between the maximum and minimum values of various indicator data in the past 13 years. Among the input indicators, the standard deviation of AB was the largest at 18,701.15. In the output indicators, DT had the highest standard deviation of 1873.41. Among environmental variables, the FTPS standard deviation was the highest at 17,139.75 (Table 1). MHI, AB, number of health workers (HW), PAP, and RN were selected for fairness analysis.

**Table 1:** Descriptive analysis of input–output and environmental variables.

Primary indicators	Secondary indicators	Abbreviation	Mean	SD	Min	Max
Input indicators	Medical and health institutions (unit)	MHI	1761.50	2134.82	71.00	12497.00
	Actual beds (number)	AB	12810.35	18701.15	616.00	160833.00
	Practicing (assistant) physicians (person)	PAP	4958.04	8261.34	568.00	80002.00
	Registered nurses (person)	RN	5310.34	9970.34	333.00	100742.00
Output indicators	Number of diagnoses and treatments (10,000 person times)	DT	1134.90	1873.41	67.20	16451.64
	Number of surgeries performed (10,000 person times)	SP	8.72	16.12	0.60	172.13
	Discharged patients (10,000 persons)	DP	39.84	56.67	2.22	479.34
Environment variables	Gross domestic product (100 million yuan)	GDP	992.24	1811.03	65.71	19916.98
	Health expenditure (100 million yuan)	HE	15.23	19.40	0.55	187.64
	Permanent residents (10,000 persons)	PR	214.92	260.10	27.72	2119.20
	Full-time teachers in primary and secondary schools (person)	FTPS	15608.28	17139.75	1773.00	125365.00
	Urbanization rate (%)	UR	57.83	19.35	28.89	100.00
	General public budget revenue (100 million yuan)	GPBR	67.92	162.35	2.54	1697.63

## 3 Results

### 3.1 Current situation of health resource allocation in Chengdu–Chongqing Economic Circle

From 2009 to 2021, although the number of MHI decreased in 2016, 2020, and 2021, it showed an overall growth trend. The number of AB, HW, PAP, and RN were increasing each year. The average annual growth rates of MHI, AB, HW, PAP, and RN were 2.24, 7.98, 6.51, 5.65, and 10.79%, respectively. Apart from MHI, there has been an annual increase in AB, HW, PAP, and RN per thousand population and per square kilometer (Table 2).

**Table 2:** Health resource allocation in Chengdu–Chongqing Economic Circle from 2009 to 2021.

Year	MHI			AB			HW			PAP			RN		
	/1000 persons	/km^**2**^	Total	/1000 persons	/km^**2**^	Total	/1000 persons	/km^**2**^	Total	/1000 persons	/km^**2**^	Total	/1000 persons	/km^**2**^	Total
2009	0.6740	0.3061	62,459	3.3315	1.5128	308,734	5.1413	2.3347	476,453	1.6736	0.7600	155,099	1.1654	0.5292	107,995
2010	0.7947	0.3560	72,659	3.8068	1.7055	348,056	5.8630	2.6267	536,055	1.8051	0.8087	165,040	1.3777	0.6172	125,961
2011	0.8077	0.3626	74,199	4.2199	1.8942	387,638	6.3070	2.8310	579,351	1.8975	0.8518	174,306	1.5785	0.7085	145,000
2012	0.8071	0.3584	74,473	4.8634	2.1599	448,777	6.8137	3.0260	628,746	2.0137	0.8943	185,822	1.8145	0.8058	167,438
2013	0.8421	0.3774	78,207	5.3260	2.3870	494,614	7.3107	3.2764	678,924	2.1311	0.9551	197,912	2.0155	0.9033	187,172
2014	0.8450	0.3807	78,897	5.7045	2.5704	532,624	7.6750	3.4583	716,610	2.1952	0.9891	204,964	2.2407	1.0096	209,210
2015	0.8415	0.3824	79,236	6.0543	2.7511	570,065	7.9508	3.6129	748,642	2.2306	1.0136	210,029	2.4234	1.1012	228,186
2016	0.8307	0.3806	78,876	6.4052	2.9350	608,174	8.2446	3.7778	782,818	2.2850	1.0470	216,957	2.6261	1.2033	249,351
2017	0.8317	0.3837	79,511	6.8828	3.1756	658,026	8.6716	4.0009	829,041	2.4025	1.1085	229,692	2.8695	1.3239	274,333
2018	0.8460	0.3926	81,352	7.2743	3.3757	699,493	9.1127	4.2289	876,276	2.5586	1.1874	246,036	3.1147	1.4454	299,509
2019	0.8634	0.4029	83,496	7.6353	3.5635	738,402	9.6467	4.5023	932,928	2.7594	1.2878	266,856	3.3889	1.5816	327,736
2020	0.8392	0.3993	82,743	7.6833	3.6560	757,569	9.8760	4.6994	973,774	2.8737	1.3674	283,346	3.5110	1.6707	346,184
2021	0.8254	0.3932	81,470	7.8550	3.7422	775,347	10.2922	4.9034	1,015,923	3.0387	1.4477	299,940	3.7427	1.7831	369,439

### 3.2 The equity of health resource allocation in Chengdu–Chongqing Economic Circle

**Figure 2:**
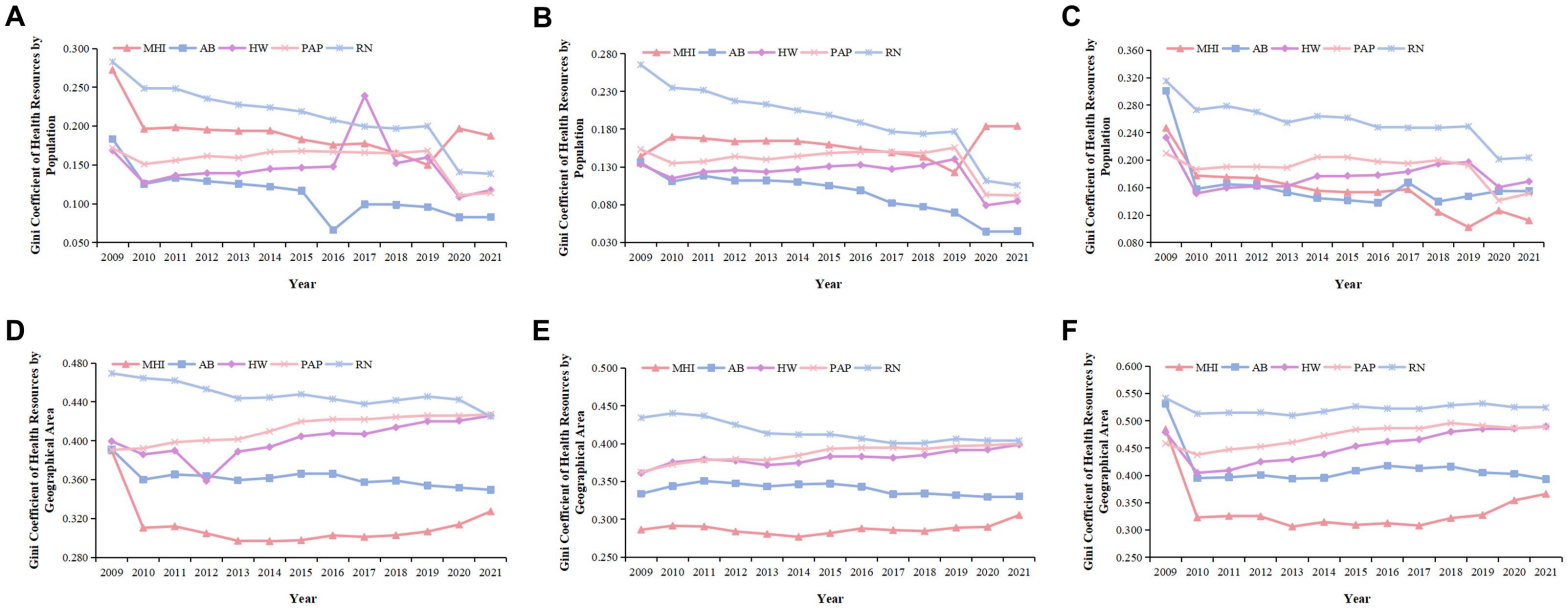
Gini coefficient of health resource allocation in Chengdu–Chongqing Economic Circle from 2009 to 2021. **(A,D)** Are the Gini coefficients of health resources in Chengdu-Chongqing Economic Circle allocated by population and geographical area, respectively. **(B,E)** Are the Gini coefficients of health resources in Chengdu Economic Circle allocated by population and geographical area, respectively. **(C,F)** Are the Gini coefficients of health resources in Chongqing Economic Circle allocated by population and geographical area, respectively.

From 2009 to 2021, according to population allocation, the Gini coefficient of various health resources in Chengdu-Chongqing Economic Circle showed a fluctuating downward trend, indicating that the fairness of health resources has improved after the new medical reform. The evolution trend of the Gini coefficient of HW showed an “inverted V-shape,” reaching its peak in 2017 at 0.239. In contrast, the Gini coefficient of AB displayed a “V-shaped” trend, reaching a trough of 0.066 in 2016. The Gini coefficient of most health resources allocated by population was less than 0.2, suggesting absolute equity. From a regional perspective, the Gini coefficients of various health resources in Chengdu Economic Circle ranged from 0.044 to 0.265. Except for RN, the Gini coefficients of all other health resources were less than 0.2. For Chongqing Economic Circle, the Gini coefficients of various health resources ranged from 0.102 to 0.315. Except for 2009, the Gini coefficients of MHI, AB, and HW were less than 0.2 (Figures 2A–C).

When configured by geographical area, the Gini coefficients of AB and RN in Chengdu-Chongqing Economic Circle have shown an overall downward trend in the past 13 years, while the Gini coefficients of MHI, HW, and PAP have shown an overall upward trend. Moreover, the Gini coefficients of most health resources ranged from 0.3 to 0.5, which was between relatively reasonable and relative inequity. From 2009 to 2010, MHI experienced a greater decrease in its Gini coefficient than other health resources, with a decrease of 0.079. The fairness of RN was even worse. From a regional perspective, the Gini coefficients of various health resources in Chengdu Economic Circle did not change much, with MHI, AB, HW, and PAP having Gini coefficients ranging from 0.277 to 0.400, while the Gini coefficients of RN ranged from 0.401 to 0.440. The Gini coefficient values of various health resources in Chongqing Economic Circle ranged from 0.306 to 0.542. Furthermore, the decline in various health resources was larger from 2009 to 2010, but remained relatively stable in other periods (Figures 2D–F).

The Theil index and Gini coefficient of health resource allocation in Chengdu-Chongqing Economic Circle showed a generally consistent evolution trend (31). Further analysis of the sources of unfairness revealed that the main reason for the inequity in health resource allocation in Chengdu-Chongqing Economic Circle was intra-regional differences. From 2009 to 2021, the contribution rates of AB, HW, PAP, and RN within the region were greater than those between regions, and the intra-regional contribution rates exceeded 94%. Except for the relatively small intra-regional contribution rate of MHI in 2009, the intra-regional contribution rate of MHI in all other years was greater than the inter-regional contribution rate (Table 3).

**Table 3:** Theil index of health resource allocation in Chengdu-Chongqing Economic Circle.

Year	Theil index					Contribution rate of intra-region(%)					Contribution rate of inter-region(%)				
	MHI	AB	HW	PAP	RN	MHI	AB	HW	PAP	RN	MHI	AB	HW	PAP	RN
2009	0.0569	0.0272	0.0237	0.0225	0.0614	29.63	99.53	94.65	98.79	99.66	70.37	0.47	5.35	1.21	0.34
2010	0.0262	0.0135	0.0154	0.0186	0.0490	79.47	99.98	100.00	99.85	99.77	20.53	0.02	0.00	0.15	0.23
2011	0.0265	0.0146	0.0177	0.0199	0.0497	76.64	99.71	99.63	98.95	99.99	23.36	0.29	0.37	1.05	0.01
2012	0.0259	0.0133	0.0183	0.0205	0.0456	76.08	98.48	99.20	97.91	100.00	23.92	1.52	0.80	2.09	0.00
2013	0.0254	0.0125	0.0182	0.0204	0.0420	75.92	99.38	98.67	97.34	99.97	24.08	0.62	1.33	2.66	0.03
2014	0.0255	0.0118	0.0199	0.0233	0.0414	72.76	99.48	98.95	98.04	99.99	27.24	0.52	1.05	1.96	0.01
2015	0.0226	0.0110	0.0211	0.0237	0.0402	77.20	99.91	99.83	98.91	99.98	22.80	0.09	0.17	1.09	0.02
2016	0.0210	0.0104	0.0213	0.0231	0.0361	77.17	99.97	99.99	99.50	99.96	22.83	0.03	0.01	0.50	0.04
2017	0.0216	0.0087	0.0215	0.0229	0.0347	72.66	99.71	99.89	99.38	100.00	27.34	0.29	0.11	0.62	0.00
2018	0.0184	0.0089	0.0227	0.0229	0.0336	72.14	99.56	99.97	99.99	99.82	27.86	0.44	0.03	0.01	0.18
2019	0.0153	0.0085	0.0245	0.0235	0.0348	64.99	99.26	99.88	100.00	99.93	35.01	0.74	0.12	0.00	0.07
2020	0.0264	0.0089	0.0161	0.0136	0.0221	78.85	98.27	99.80	100.00	99.92	21.15	1.73	0.20	0.00	0.08
2021	0.0246	0.0087	0.0174	0.0141	0.0220	82.30	97.91	99.21	99.72	99.96	17.70	2.09	0.79	0.28	0.04

We further decomposed the intra-regional differences. From 2009 to 2021, the contribution rate of differences in MHI allocation in Chengdu Economic Circle showed an upward trend, whereas the contribution rate of differences in the allocation of other health resources showed an overall downward trend. The Chongqing Economic Circle was the opposite. Before 2018, the internal differences in Chongqing Economic Circle contributed more to the differences in MHI allocation. However, the internal differences in Chengdu Economic Circle have contributed more to the differences in MHI allocation since 2018. The internal differential contribution rates of AB, HW, PAP, and RN allocation in Chongqing Economic Circle were greater than 71, 72, 67, and 62% respectively, indicating that the inequity of AB, HW, PAP, and RN mainly comes from Chongqing Economic Circle (Table 4).

**Table 4:** Proportion of disparities in contribution between Chengdu Economic Circle and Chongqing Economic Circle.

Year	MHI		AB		HW		PAP		RN	
	Chengdu Economic Circle	Chongqing Economic Circle	Chengdu Economic Circle	Chongqing Economic Circle	Chengdu Economic Circle	Chongqing Economic Circle	Chengdu Economic Circle	Chongqing Economic Circle	Chengdu Economic Circle	Chongqing Economic Circle
2009	24.03	75.97	15.41	84.59	22.34	77.66	28.67	71.33	37.43	62.57
2010	43.73	56.27	22.85	77.15	27.05	72.95	26.23	73.77	35.96	64.04
2011	43.60	56.40	24.96	75.04	26.74	73.26	25.97	74.03	33.48	66.52
2012	42.49	57.51	24.43	75.57	26.99	73.01	28.93	71.07	31.77	68.23
2013	45.37	54.63	26.99	73.01	25.16	74.84	27.71	72.29	33.08	66.92
2014	48.01	51.99	28.04	71.96	23.91	76.09	25.22	74.78	30.44	69.56
2015	48.33	51.67	27.39	72.61	24.61	75.39	27.37	72.63	29.70	70.30
2016	47.13	52.87	24.78	75.22	24.54	75.46	29.52	70.48	29.25	70.75
2017	44.48	55.52	18.90	81.10	21.88	78.12	29.85	70.15	26.33	73.67
2018	54.71	45.29	15.54	84.46	22.49	77.51	28.74	71.26	26.45	73.55
2019	55.56	44.44	12.67	87.33	24.09	75.91	32.11	67.89	26.67	73.33
2020	66.35	33.65	4.95	95.05	8.75	91.25	15.64	84.36	13.32	86.68
2021	70.05	29.95	5.38	94.62	9.54	90.46	14.99	85.01	11.82	88.18

**Figure 3:**
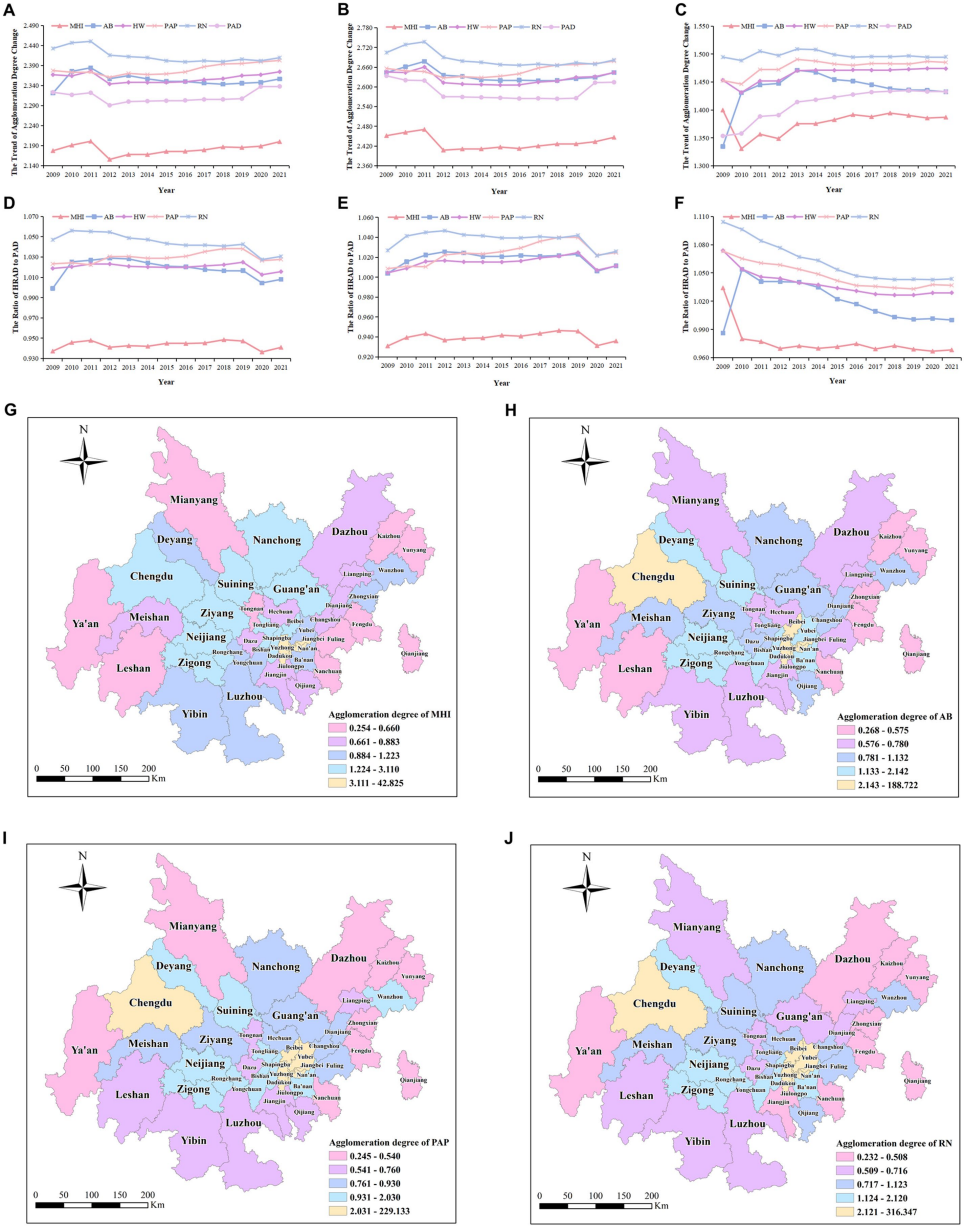
Agglomeration degree in Chengdu-Chongqing Economic Circle from 2009 to 2021. **(A–C)** represent the HRAD and PAD in Chengdu-Chongqing Economic Circle, Chengdu Economic Circle, and Chongqing Economic Circle, respectively. **(D–F)** Represent the ratios of HRAD to PAD in Chengdu-Chongqing Economic Circle, Chengdu Economic Circle, and Chongqing Economic Circle, respectively. **(G–J)** represent the agglomeration degree of MHI, AB, PAP, and RN in various districts of Chengdu-Chongqing Economic Circle.

The agglomeration degree of various health resources in Chengdu-Chongqing Economic Circle from 2009 to 2021 exceeded 2.140. This result denotes that the fairness of health resource allocation based on geographical area is relatively high. The agglomeration degree of RN was the highest, while MHI’s was the lowest but greater than 1, which indicates that RN is more concentrated compared to other health resources. From a regional perspective, the HRAD of Chengdu Economic Circle exceeded 2.360, higher than the average level, showing a relative surplus of health resources. The HRAD in Chongqing Economic Circle exceeded 1.300, indicating a relative concentration of health resources (Figures 3A–C).

The agglomeration ratios of various health resources in Chengdu-Chongqing Economic Circle were relatively stable, with the ratios of AB, HW, PAP, and RN basically greater than 1, while the ratios of MHI were all less than 1. The results suggest that apart from MHI, most health resources are allocated fairly by population. The trend of changes in the ratio of various health resources in Chengdu Economic Circle was the same as that in Chengdu-Chongqing Economic Circle, whereas the overall ratios of various health resources in Chongqing Economic Circle exhibited a downward trend. The ratio of MHI of Chongqing Economic Circle decreased from 1.034 in 2009 to 0.968 in 2021, and the ratio of AB fluctuated around 1, while the ratios of other health resources were all greater than 1. This suggests that the health human resources in Chongqing Economic Circle are sufficient relative to the population (Figures 3D–F).

Looking at different districts, in terms of MHI, the agglomeration degree of most districts (63.64%) was below 1.224. The agglomeration degree of 10 districts including Chengdu and Tongliang ranged from 1.224 to 1.933. The agglomeration degree of six main urban districts in Chongqing Economic Circle, including Yuzhong and Dadukou, was greater than 3, with Yuzhong reaching a high of 42.825. Regarding AB, the agglomeration degree in most districts (61.36%) was below 1.133. The agglomeration degree of eight districts including Chengdu and Shapingba was greater than 2.142, especially in Yuzhong, which was as high as 188.722. Regarding human resources, the agglomeration degree of PAP and RN in over half of the districts was below 0.931. The agglomeration degree of human resources in nine districts including Chengdu and Yubei was greater than 2.031. PAP and RN agglomeration degrees in Yuzhong were 229.133 and 316.347, respectively. These results suggest the health resources in Chengdu-Chongqing Economic Circle are concentrated in the core areas, showing a significant disparity in the distribution of health resources among different districts (Figures 3G–J).

### 3.3 HRAE in Chengdu–Chongqing Economic Circle

#### 3.3.1 Efficiency analysis based on the traditional DEA model in the first stage

Research has shown that the average TE, SE, and PTE of the Chengdu-Chongqing Economic Circle from 2009 to 2021 were 0.841, 0.920, and 0.914, respectively. Compared to 2009, the TE of the Chengdu-Chongqing Economic Circle in 2021 has increased. The Chengdu Economic Circle has increased by 0.409, and the Chongqing Economic Circle has increased by 0.091, which denotes more room for improvement in Chengdu Economic Circle than in Chongqing Economic Circle. Compared with the 12th Five-Year Plan period, the TE of the Chengdu-Chongqing Economic Circle increased during the 13th Five-Year Plan period by 0.036 in Chengdu Economic Circle and 0.021 in Chongqing Economic Circle. In 2021, 12 districts, including Deyang and Yuzhong, were in a DEA effective state, 12 districts, including Chengdu and Dadukou, were in a DEA weakly effective state, and 20 districts, including Suining and Fuling, were in a DEA ineffective state. Among the 15 districts in Chengdu Economic Circle, 2 districts (13.33%) had DEA effectiveness, 8 districts (53.33%) had DEA weak effectiveness, and 5 districts (33.33%) had DEA inefficiency. Among the 29 districts in Chongqing Economic Circle, 10 districts (34.48%) had DEA effectiveness, 4 districts (13.79%) had weak DEA effectiveness, and 15 districts (51.72%) had DEA inefficiency (Appendix Table 1).

**Figure 4:**
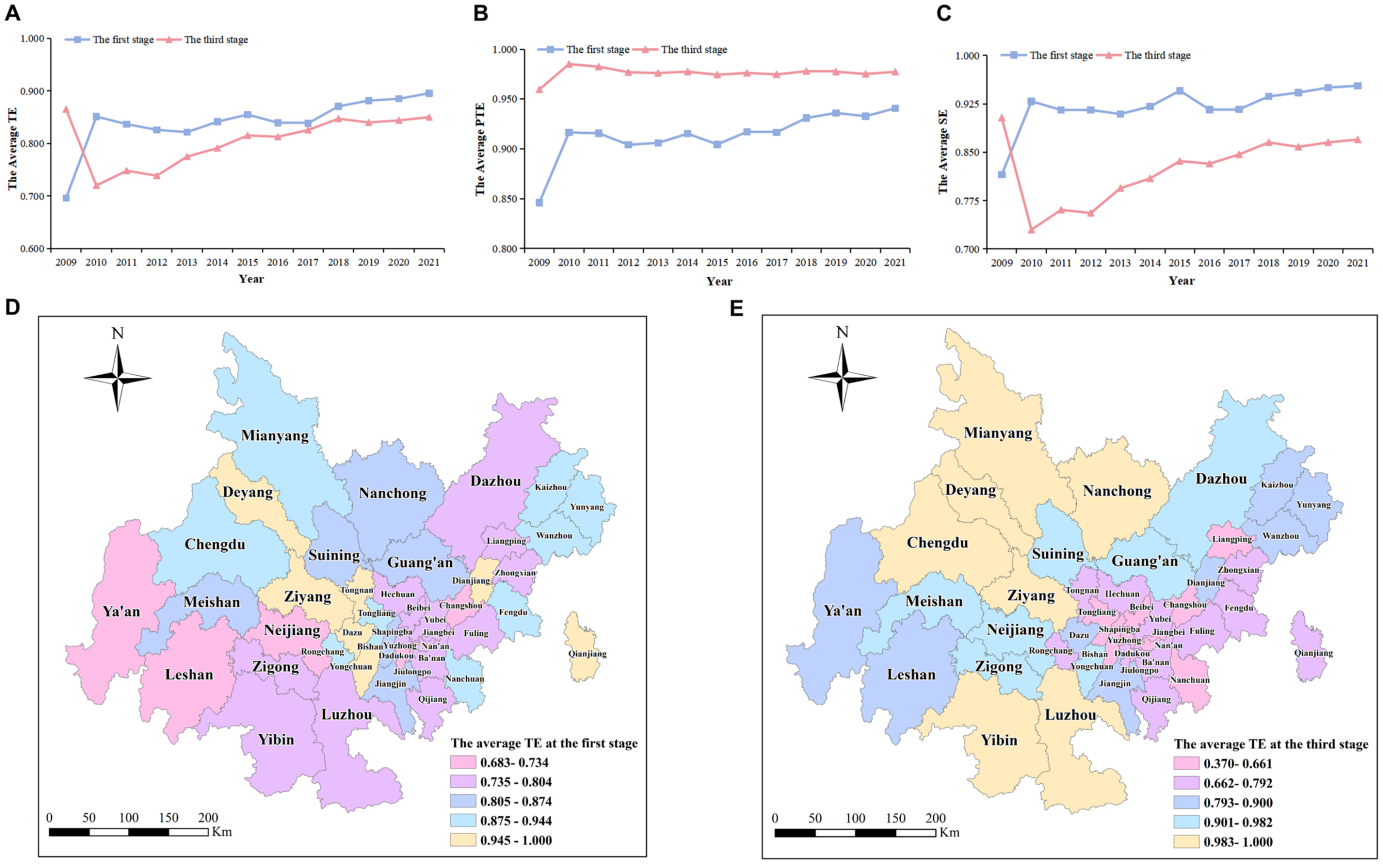
Comparison of HRAE in the first and third stages of Chengdu-Chongqing Economic Circle from 2009 to 2021. **(A–C)** signify the average TE, PTE, and SE of health resource allocation in Chengdu-Chongqing Economic Circle at stage 1 and stage 3, respectively. **(D,E)** Signify the average TE of each district in Chengdu-Chongqing Economic Circle at stage 1 and stage 3, respectively.

From 2009 to 2021, TE, SE, and PTE in Chengdu-Chongqing Economic Circle showed a fluctuating upward trend, with a more significant increase from 2009 to 2010. The trend of SE and TE changes was generally consistent, indicating that SE is the main factor affecting TE changes. Looking at different districts, from 2009 to 2021, the TE of most districts (61.36%) was less than 0.875, whereas the TE of eight districts, including Deyang and Tongnan, was greater than 0.945. Yuzhong and Qianjiang reached the DEA effective state. A second stage SFA regression analysis is required to obtain the true HRAE (Figures 4A–D).

#### 3.3.2 Analysis of results based on the SFA regression model in the second stage

This article used Frontier 4.1 to construct the SFA regression model with environmental variables as independent variables and input slack values as dependent variables. All four input slack variables passed the LR test at the 1% level, and the corresponding σ^2^ and γ also passed the t-test at the 1% level. Therefore, removing environmental variables and random disturbances is reasonable (16). Additionally, γ ≥ 0.68 exhibits that HRAE is mainly affected by management inefficiency (32) (Table 5).

**Table 5:** Analysis of regression results based on SFA model at the second stage.

Variables	MHI slack		AB slack		PAP slack		RN slack	
	Coefficient	***T*-value**	Coefficient	***T*-value**	Coefficient	***T*-value**	Coefficient	***T*-value**
Constant term	55.38^***^	3.97	426.81^**^	2.03	175.16^*^	1.74	139.23^*^	1.76
GDP	0.01	0.33	0.17	1.16	0.18^**^	2.57	0.12^*^	1.91
HE	−1.57	−1.57	−13.19^***^	−3.41	−6.55^***^	−3.61	−3.88^**^	−2.43
PR	0.56^**^	2.26	2.99^***^	2.71	1.38^***^	2.81	1.16^**^	2.48
FTPS	−62.08^*^	−1.70	−316.31^*^	−1.82	−153.71^*^	−1.99	−141.31^*^	−1.94
UR	−1.34^***^	−2.94	−7.37^**^	−2.14	−3.26^**^	−2.05	−2.64^**^	−2.08
GPBR	−0.25	−0.64	−2.32	−1.47	−2.04^***^	−2.82	−1.48^**^	−2.23
σ^2^	96180.95^***^	96173.59	1956769.20^***^	1942256.90	386615.78^***^	382302.98	373468.85^***^	368561.41
γ	0.68^***^	34.30	0.76^***^	50.87	0.74^***^	44.87	0.78^***^	57.13
LR-value	248.15^***^		334.12^***^		298.66^***^		361.28^***^	

*, **, and *** Denote significance at 10, 5, 1% significance levels, respectively.

The impact coefficients of PR were all positive and significant at least under the 5% significance level. Such values indicate that the HRAE of the Chengdu-Chongqing Economic Circle decreases as PR increases. This means that increasing PR will drive the demand for medical services among residents, increasing redundant investment in health workforce and material resources and negatively affecting HRAE. The regression coefficients of UR were all negative and passed the t-test at the 5% level, indicating that the improvement of UR has a promoting effect on HRAE. The reason may be that districts with higher UR are more likely to have high-quality medical resources and services, and people can avail high-quality and convenient medical services more. The impact coefficients of HE to medical and health human and material input slack variables were all negative, and the impact coefficients to AB and PAP slack variables were significant under the 1% significance level. The results denote that an increase in HE will reduce the redundancy of health resource investment and improve HRAE. After investigating the reasons, local governments have continuously emphasized the optimization of health resource allocation (33) and increased funding and regulatory efforts in the health field since the new healthcare reform, promoting the improvement of HRAE. The regression coefficients of GPBR to various input slack variables were all negative, and the regression coefficients of GPBR to PAP and RN slack variables were significant, at least under the 5% level. That is, the increase in GPBR will positively impact HRAE. A possible reason is that local governments with more GPBR are more likely to arrange fiscal expenditures reasonably based on the health needs of residents (34). The increase in FTPS will reduce the slack values of each input variable and improve HRAE. The strengthening of the teaching staff may promote the development of education and broaden the coverage of the population receiving education, which enable people to make more reasonable use of health resources and thereby improve the utilization rate of health resources. Moreover, the increase in GDP negatively impacts HRAE, and the impact coefficient of GDP to PAP slack variable was significant at the 5% level. Considering the continuous promotion of the construction of the Chengdu-Chongqing Economic Circle, the economy has been continuously developing. At the same time, people’s purchasing power has been constantly improving, and investment in healthcare has been increasing, leading to resource waste.

#### 3.3.3 Efficiency analysis after adjusting investment indicators in the third stage

Research has shown that the average values of TE, SE, and PTE after the adjustment of the Chengdu-Chongqing Economic Circle from 2009 to 2021 were 0.806, 0.825, and 0.976, respectively. TE and SE decreased by 0.035 and 0.095 respectively, whereas PTE increased by 0.062. Thus, the low SE is the main reason for the low TE. After three-stage DEA adjustment, the TE of the Chengdu-Chongqing Economic Circle in 2021 decreased by 0.015 compared to 2009. The Chengdu Economic Circle increased by 0.031, whereas the Chongqing Economic Circle decreased by 0.038. The TE of the Chengdu-Chongqing Economic Circle during the 12th Five-Year Plan period and the 13th Five-Year Plan period decreased by 0.062 and 0.029, respectively. The TE of the 13th Five-Year Plan period was higher than that of the 12th Five-Year Plan period. Thus, not considering the external environment will overestimate the TE of these two periods. In terms of various districts, in 2021, TE increased in 17 districts including Luzhou and Jiangjin, decreased in 22 districts including Yubei and Changshou, and remained unchanged in 5 districts including Deyang and Kaizhou. After adjustment, seven districts including Bishan and Tongnan were no longer at the forefront of technology. In contrast, three new districts, including Chengdu, Mianyang, and Meishan, were at the forefront of technology (Appendix Table 1).

From 2009 to 2021, the adjusted TE and SE showed a trend of first decreasing and then increasing, whereas PTE showed a trend of first increasing and then steadily decreasing. TE and SE experienced a greater decline from 2009 to 2010, and their trends were generally consistent, further indicating that SE is the main influencing factor of TE changes. PTE experienced a greater increase from 2009 to 2010 and peaked in 2010. Looking at different districts, from 2009 to 2021, the TE of eight districts including Luzhou and Nanchong was greater than 0.983. The efficiency values of Chengdu and Yuzhong were all 1, reaching the DEA effective state. The rise in Neijiang and Dazhou was more pronounced, whereas the decline in Dadukou and Qianjiang was more pronounced. This indicates that environmental factors have a greater impact on these districts (Figures 4A–C,E).

### 3.4 Productivity of health resource allocation in Chengdu–Chongqing Economic Circle

Based on the adjusted input indicators and original output indicator data, the dynamic HRAE of the Chengdu-Chongqing Economic Circle was calculated again using DEAP 2.1 software. The Tfpch of the Chengdu-Chongqing Economic Circle from 2009 to 2021 was 1.027, except for 2017–2018 and 2019–2020, and the Tfpch of all other periods was greater than 1. The results show that the HRAE has shown an overall upward trend since the new healthcare reform, with an average annual increase of 2.7%. At the same time, Techch had an average annual growth of 2.8%, whereas Effch had an average annual decrease of 0.1%, indicating that the improvement of Tfpch is mainly because of the improvement of Techch. From 2009 to 2010, the Tfpch was 1.135, with a significant annual increase, which is due to the government’s high attention and determination to deepen the reform of the medical and health system, which has led to a rapid increase in Techch and thereby driven the improvement of Tfpch. From 2019 to 2020, Tfpch was 0.899, with a large average annual decline. A possible reason is that, due to the impact of COVID-19 in this period, the work focused on the prevention and control of infectious diseases, which slowed down the improvement speed of Techch, and then led to the decline of Tfpch. The frequency distribution shows that the Tfpch in 39 districts was greater than 1 from 2020 to 2021, indicating that the HRAE was developing in a good trend at the beginning of the 14th Five-Year Plan. Compared to 2009–2010, the frequency distribution of Effch (score > 1) from 2020 to 2021 was higher, while the frequency distribution of Techch (score > 1) was lower (Table 6).

**Table 6:** Dynamic HRAE and frequency distribution in Chengdu-Chongqing Economic Circle.

Year	Effch	Techch	Pech	Sech	Tfpch
2009–2010	0.797	1.424	1.028	0.776	1.135
2010–2011	1.048	1.004	0.997	1.051	1.052
2011–2012	0.991	1.124	0.994	0.997	1.114
2012–2013	1.060	0.961	0.999	1.061	1.018
2013–2014	1.028	0.985	1.002	1.027	1.012
2014–2015	1.037	0.968	0.997	1.040	1.004
2015–2016	0.996	1.011	1.002	0.994	1.007
2016–2017	1.017	1.015	0.998	1.019	1.033
2017–2018	1.027	0.954	1.003	1.024	0.980
2018–2019	0.991	1.032	0.999	0.991	1.023
2019–2020	1.009	0.891	0.997	1.011	0.899
2020–2021	1.009	1.057	1.002	1.007	1.067
Mean	0.999	1.028	1.002	0.997	1.027
Frequency distribution (2009–2010)					
>1	12	44	23	7	23
1	3	0	12	5	0
<1	29	0	9	32	21
Frequency distribution(2020–2021)					
>1	21	43	15	19	39
1	6	0	20	6	0
<1	17	1	9	19	5

In terms of Tfpch, except for the three districts of Jiangjin, Hechuan, and Tongnan where Tfpch was less than 1 and Fengdu where the Tfpch was equal to 1, the Tfpch in all other districts was greater than 1. Tfpch in Qianjiang was the highest at 1.071, whereas Tfpch in Jiangjin was the lowest at 0.983. In terms of Effch, 16 districts (36.36%) had an Effch greater than 1, 25 districts (56.82%) less than 1, and 3 districts (6.82%) had it equal to 1. In terms of Techch, except for the three districts of Jiangjin, Tongnan, and Zhongxian, where Techch was less than 1, all other districts were greater than 1 (Appendix Table 2).

## 4 Discussion

Since the new healthcare reform in 2009, how to allocate health resources reasonably has been a hot topic of discussion. The Planning Outline attaches great importance to the development of the Chengdu-Chongqing Economic Circle, placing it at an important level with the Yangtze River Delta, the Guangdong-Hong Kong-Macao Greater Bay Area, and the Beijing-Tianjin-Hebei at the national strategic level. It also emphasizes the importance of promoting the sinking of high-quality medical resources and improving the two-way referral mechanism. Optimizing the allocation of medical resources is an important connotation of promoting integrated health and hygiene development in Chengdu-Chongqing Economic Circle. However, equity and efficiency are very important and difficult to balance when allocating medical resources. Therefore, this study empirically analyzes the health resource allocation-related issues encountered in constructing the Chengdu-Chongqing Economic Circle.

Since the new healthcare reform, the total amount of health resources in Chengdu-Chongqing Economic Circle, as well as health resources per thousand people and per square kilometer, have steadily increased over the past 13 years. Compared to other health resources, the growth rate of MHI is relatively small. Given the changes in the development mode of public hospitals and the allocation of health resources in recent years, the expansion of the scale of public hospitals has been limited to some extent (35). Simultaneously, as a critical component of MHI, public hospitals have also affected the growth rate of MHI. This study uses the Gini coefficient, Theil index, and agglomeration degree to study the fairness of health resource allocation in Chengdu-Chongqing Economic Circle. The empirical results show that the Gini coefficient range of each health resource in Chengdu-Chongqing Economic Circle according to population allocation is 0.066–0.283, while the Gini coefficient range for geographical area allocation is 0.297–0.469. The results denote that the fairness of health resources allocated by geographical area is worse than that allocated by population, which is consistent with existing research results (36). Possible reasons for this situation include the government’s goal of fulfilling residents’ health service needs and the impact of economic disparities in different districts of the Chengdu-Chongqing Economic Circle on the health resource allocation. The distance between residents and MHI should not be ignored as it can affect their convenience and enthusiasm for seeking medical treatment. Therefore, when formulating health plans, the government should comprehensively consider population and geographical area factors (9, 37). At the same time, the Gini coefficient of health resources allocated by population in Chengdu-Chongqing Economic Circle shows a downward trend. The equity of population allocation is constantly improving, which is closely related to the government’s emphasis on health resource allocation, and may also be related to changes in population structure and the health needs of residents. For the older adult and those with chronic diseases, the service capacity of primary-level medical and health institutions should be strengthened to make basic medical and health services more fair and accessible.

The research results indicate that intra-regional differences are the main reason for the unfair allocation of health resources in Chengdu-Chongqing Economic Circle. The contribution rate of internal differences in the allocation of most health resources in Chongqing Economic Circle is greater than that in Chengdu Economic Circle, which means larger internal differences are found in the allocation of health resources in Chongqing Economic Circle. Further analysis reveals that the reasons for the differences in the allocation of health resources in different regions include the level of economic development and the geographical distribution of MHI. Economically developed regions usually provide more medical resources and higher quality medical services, while remote areas have a weak attraction to health talents, resulting in insufficient high-quality medical resources and lower medical service capabilities. We must first solve the problem of unequal distribution of health resources in Chongqing Economic Circle, and lay a good foundation for scientific expansion of high-quality medical resources and regional balanced distribution of high-quality medical resources in Chengdu-Chongqing Economic Circle. The government should pay attention to the internal differences in the allocation of health resources in Chongqing Economic Circle, provide financial support to economically underdeveloped and remote areas such as Qianjiang, and promote the construction of close county-level medical communities. This suggestion aims to enhance the accessibility of high-quality medical resources, establish a high-quality and efficient integrated medical and health service system, and meet the health needs of residents in remote areas.

The fairness of most health resources in Chengdu-Chongqing Economic Circle based on population and geographical area allocation is good, but the fairness of MHI based on population allocation needs to be further improved. This result is similar to that of Yixin et al. (38). Research has shown that the regional agglomeration of health resources has been observed in Chengdu-Chongqing Economic Circle, with health resources mainly concentrated in economically developed core areas, consistent with existing research results basically (39, 40). Considering the region’s small geographical area and relatively developed economy, health resources are tilted toward the region, presenting a “Matthew effect” in terms of health resource allocation (41). Additionally, the characteristics of health resource allocation are largely consistent with the trend of urbanization, spreading from the core area outwards (42). Thus, it’s necessary to expand the radiation scope and enhance the core areas’ driving role in health resource allocation. This will boost health resource supply in less developed areas like Yunyang and Ya’an. Simultaneously, learn relevant experiences from Beijing-Tianjin-Hebei. Districts with relatively scarce health resources can precisely connect high-quality medical resources in core areas through collaborative construction and technological exchange.

From the perspective of HRAE in Chengdu-Chongqing Economic Circle, after removing the influence of environmental factors and random interference, the HRAE in most districts of the Chengdu-Chongqing Economic Circle changed. A three-stage DEA model for efficiency analysis must be used to obtain the actual efficiency value. The efficiency analysis results showed that the adjusted average TE value of the Chengdu-Chongqing Economic Circle was 0.806 from 2009 to 2021, which was higher than India (0.655) (43), Shanxi (0.675), and Inner Mongolia (0.730) (44) but lower than the national average (0.838) (45), Iraq (0.910) (46), and the Yangtze River Delta region (0.961) (47). Before and after the adjustment, the TE during the 13th Five-Year Plan period was higher than that during the 12th Five-Year Plan period, which resulted from the government’s reforms over the years. Moreover, we found that the overall HRAE of Chengdu Economic Circle was higher than that of Chongqing Economic Circle, and the resource allocation of Chongqing Economic Circle needs further optimization. Although the government continues to promote the integrated development of health and hygiene in Chengdu-Chongqing Economic Circle, the effectiveness of the cross-regional allocation of medical resources is not significant. Further improvement of relevant supporting systems and implementation of relevant measures should be carried out. We also found that 6 districts (40.00%) in Chengdu Economic Circle, including Luzhou and Nanchong, should reduce their scale, whereas 26 districts (89.66%) in Chongqing Economic Circle, including Fuling and Jiangbei, should increase their scale. Hence, the Chengdu-Chongqing Economic Circle should establish specialized alliances and strengthen the construction of closely-integrated urban medical groups to achieve differentiated development among institutions. In addition, the Yangtze River Delta region has taken the lead in making beneficial explorations in “Internet plus healthcare.” The Chengdu-Chongqing Economic Circle should actively learn from its experience in telehealth and intelligent hospital construction and address the uneven distribution of health resources through the development of telehealth. For example, by providing online paid diagnostic and treatment services through a telemedicine platform, this measure can moderately reduce the medical burden on patients, and also reduce the service pressure of offline hospitals and the diagnostic and treatment pressure on doctors (48). Online paid diagnosis and treatment can divert excessive medical resources, allowing patients in remote areas to enjoy high-quality medical services. In addition, through cross-regional medical information sharing, doctors can make accurate diagnoses more quickly, reduce repeated examinations, save time and costs, and to some extent alleviate the gap in urban and rural medical levels (49). Health managers, by analyzing patient data, can better understand the health status and needs of various groups in different areas, thereby optimizing the allocation of medical resources (50).

Research has found spatial heterogeneity in efficiency within and between regions in Chengdu-Chongqing Economic Circle. The government should implement localized and targeted strategies for each district, considering the actual conditions of different districts to make scientific assessments. Districts such as Dadukou, where low efficiency in scale results in overall inefficiency, should be provided with preferential policies with district’s characteristics, fiscal support, and a rational expansion of scale to reduce the differences between districts. The study also found that environmental variables have a certain impact on HRAE. HE, FTPS, UR, and GPBR positively impact efficiency, whereas GDP and PR negatively impact efficiency. Therefore, these influencing factors should be comprehensively considered, and multiple measures should be adopted to improve HRAE. Regarding productivity, the HRAE has shown an overall upward trend since the new healthcare reform, and the improvement of Techch has driven the improvement of Tfpch. Furthermore, the Tfpch and Techch in most districts were greater than 1, whereas Effch was less than 1. Further decomposition confirms that low Sech leads to low Effch, indicating room for improvement in the Sech of the Chengdu-Chongqing Economic Circle. Therefore, MHI needs to anchor the demand of the medical service market accurately based on the local population size, economic level, and disease spectrum (51), allocate health resources scientifically and reasonably, and improve the utilization rate of health resources.

## 5 Conclusion

The Chengdu-Chongqing Economic Circle faces issues such as the need for further improvement in HRAE and Sech, imbalances in health resource allocation between regions and within different areas of the region, and significant differences in HRAE. Overall, due to Chongqing being a mega-city that integrates large urban areas, rural areas, mountainous areas, and reservoir areas, and Chengdu having advantages as a provincial capital and geographical benefits of the Chengdu Plain, the Chengdu Economic Circle has relatively better equity compared to the Chongqing Economic Circle, which is consistent with the results of the efficiency analysis. To address these issues, regional cooperation and sharing should be strengthened to improve the equity and efficiency of regional health resource allocation.

### 5.1 Limitations

Our study has certain limitations. First, this study included representative indicators of human and material resources but did not include indicators of financial resources. As a result, our findings might not completely reflect the aggregate status of health resources in Chengdu-Chongqing Economic Circle. Second, given the unavailability of the Chongqing Health Statistical Yearbook before 2009, relevant research on the early stage of the Eleventh Five-Year Plan was not conducted. Finally, our research methods mainly explored the fairness of health resources from the dimensions of population and geographical area, without fully considering the impact of the economic factors on equity. Moreover, the three-stage DEA model cannot rank DEA-efficient decision-making units, which has certain limitations.

## Data availability statement

The original contributions presented in the study are included in the article/Supplementary material, further inquiries can be directed to the corresponding author.

## Author contributions

TW: Data curation, Supervision, Writing – original draft, Writing – review & editing. TZ: Data curation, Writing – original draft. LZ: Data curation, Writing – review & editing. YH: Writing – review & editing. JW: Data curation, Writing – review & editing. YW: Writing – review & editing. LH: Supervision, Writing – review & editing.

## References

[1] Tian Y Peng J Liu Y Huang J. Efficiency trends of essential public health services and possible influencing factors since the new round health reform in China: a case study from Hainan Province. Front Public Health. (2023) 11:1269473. doi: 10.3389/fpubh.2023.1269473, PMID: 38026396 PMC10657853

[2] Yip W Fu H Chen AT Zhai T Jian W Xu R. 10 years of health-care reform in China: progress and gaps in universal health coverage. Lancet. (2019) 394:1192–204. doi: 10.1016/S0140-6736(19)32136-1 31571602

[3] Tao W Zeng Z Dang H Li P Chuong L Yue D. Towards universal health coverage: achievements and challenges of 10 years of healthcare reform in China. BMJ Glob Health. (2020) 5:e002087. doi: 10.1136/bmjgh-2019-002087, PMID: 32257401 PMC7103842

[4] Li Z Yang L Tang S Bian Y. Equity and efficiency of health resource allocation of Chinese medicine in mainland China: 2013-2017. Front Public Health. (2020) 8:579269. doi: 10.3389/fpubh.2020.579269, PMID: 33384979 PMC7769806

[5] Wu S Deng X Qi Y. Factors driving coordinated development of urban green economy: an empirical evidence from the Chengdu-Chongqing economic circle. Int J Environ Res Public Health. (2022) 19:6107. doi: 10.3390/ijerph19106107, PMID: 35627642 PMC9141902

[6] Han D Chen L Wu H Wang X Xiao Y Yang H. Evaluation on coupling coordinated development of population economy and eco-geological environment in the twin-city economic circle of Chengdu-Chongqing region. Sci Rep. (2023) 13:13459. doi: 10.1038/s41598-023-40352-w, PMID: 37596317 PMC10439235

[7] Wei L Fangjuan D Ou R. Geographical detection of the allocation and influencing factors of different healthcare resources: take the main city of Guiyang as an example. Bull Surveying Mapping. (2022) 116-120:125. doi: 10.13474/j.cnki.11-2246.2022.0366

[8] Pu L. Fairness of the distribution of public medical and health resources. Front Public Health. (2021) 9:768728. doi: 10.3389/fpubh.2021.768728, PMID: 34858935 PMC8631734

[9] Sun J Luo H. Evaluation on equality and efficiency of health resources allocation and health services utilization in China. Int J Equity Health. (2017) 16:127. doi: 10.1186/s12939-017-0614-y, PMID: 28709422 PMC5513103

[10] Zhou Z Zhu L Zhou Z Li Z Gao J Chen G. The effects of China's urban basic medical insurance schemes on the equity of health service utilisation: evidence from Shaanxi Province. Int J Equity Health. (2014) 13:23. doi: 10.1186/1475-9276-13-23, PMID: 24606592 PMC4016277

[11] Zhang T Xu Y Ren J Sun L Liu C. Inequality in the distribution of health resources and health services in China: hospitals versus primary care institutions. Int J Equity Health. (2017) 16:42. doi: 10.1186/s12939-017-0543-9, PMID: 28253876 PMC5335774

[12] Muzi L Xiaoyuan Q Yanli Z Yuanna Z Zhihui L. Analysis of the equity of health resource allocation in grassroots health care institutions in China during the 13th five-year plan period. Chin Hospitals. (2023) 27:22–5. doi: 10.19660/j.issn.1671-0592.2023.02.06

[13] Xiaoyu W Qianqian W Jingju X Yuzhuo L Zichen X Chao X. Fairness of health resource allocation. China Modern Prevent Med. (2022) 49:845–50.

[14] Zhixin F Peng Y Chao Z Ting W Jia Y Qiang S. Evaluation on the health service resources allocation efficiency in Shandong Province under hierarchical diagnosis and treatment. Chinese Health Econ. (2024) 43:42–6.

[15] Mengliu Z Qiaohui D Qi D Yue X Jing W Yi X. Research on the equity and efficiency of health resource allocation in the Yangtze River Delta region. Med Soc. (2024) 37:96. doi: 10.13723/j.yxysh.2024.04.011

[16] Jiaying W Kainuo L Qiu Z Yuqi Z Baoyi Z Siyuan X. Equity and efficiency of health resources allocation in the Greater Bay Area. China J Pharmaceut Econ. (2023) 18:33. doi: 10.12010/j.issn.1673-5846.2023.08.004

[17] Zhou M. Regional differences in health resource allocation: a longitudinal study in the Chengdu-Chongqing economic circle. China. BMJ Open. (2024) 14:e082721. doi: 10.1136/bmjopen-2023-082721, PMID: 38514148 PMC10961522

[18] Zhang Y Wang Q Jiang T Wang J. Equity and efficiency of primary health care resource allocation in mainland China. Int J Equity Health. (2018) 17:140. doi: 10.1186/s12939-018-0851-8, PMID: 30208890 PMC6134520

[19] Yang C Cui D Yin S Wu R Ke X Liu X. Fiscal autonomy of subnational governments and equity in healthcare resource allocation: evidence from China. Front Public Health. (2022) 10:989625. doi: 10.3389/fpubh.2022.989625, PMID: 36249207 PMC9561467

[20] Wang Y Li Y Qin S Kong Y Yu X Guo K. The disequilibrium in the distribution of the primary health workforce among eight economic regions and between rural and urban areas in China. Int J Equity Health. (2020) 19:28. doi: 10.1186/s12939-020-1139-3, PMID: 32102655 PMC7045560

[21] Suwei Y Fengqing W Wenwei L Zhe Z Jin M. Methodology discussion of health resource allocation equity evaluation based on agglomeration degree. Chin Hosp Manag. (2015) 35:3–5.

[22] Dai G Li R Ma S. Research on the equity of health resource allocation in TCM hospitals in China based on the Gini coefficient and agglomeration degree: 2009-2018. Int J Equity Health. (2022) 21:145. doi: 10.1186/s12939-022-01749-7, PMID: 36199086 PMC9534739

[23] Mohd Hassan NZA Bahari MS Aminuddin F Kunusagaran MNS Zaimi NA Mohd Hanafiah AN. Data envelopment analysis for ambulance services of different service providers in urban and rural areas in Ministry of Health Malaysia. Front Public Health. (2023) 10:959812. doi: 10.3389/fpubh.2022.959812, PMID: 36684911 PMC9853528

[24] Wang X Luo H Qin X Feng J Gao H Feng Q. Evaluation of performance and impacts of maternal and child health hospital services using data envelopment analysis in Guangxi Zhuang autonomous region, China: a comparison study among poverty and non-poverty county level hospitals. Int J Equity Health. (2016) 15:131. doi: 10.1186/s12939-016-0420-y, PMID: 27552805 PMC4994280

[25] Yao S Wu G. Research on the efficiency of green agricultural science and technology innovation resource allocation based on a three-stage DEA model-a case study of Anhui Province, China. Int J Environ Res Public Health. (2022) 19:13683. doi: 10.3390/ijerph192013683, PMID: 36294259 PMC9603484

[26] Wang C Zeng J Zhong H Si W. Scientific research input and output efficiency evaluation of universities in Chengdu-Chongqing economic circle based on data envelopment analysis. PLoS One. (2023) 18:e0287692. doi: 10.1371/journal.pone.0287692, PMID: 37418446 PMC10328349

[27] Liu W Xia Y Hou J. Health expenditure efficiency in rural China using the super-SBM model and the Malmquist productivity index. Int J Equity Health. (2019) 18:111. doi: 10.1186/s12939-019-1003-5, PMID: 31324184 PMC6642491

[28] Cheng J Kuang X Zeng L. The impact of human resources for health on the health outcomes of Chinese people. BMC Health Serv Res. (2022) 22:1213. doi: 10.1186/s12913-022-08540-y, PMID: 36175870 PMC9521871

[29] Lee KS Chun KH Lee JS. Reforming the hospital service structure to improve efficiency: urban hospital specialization. Health Policy. (2008) 87:41–9. doi: 10.1016/j.healthpol.2007.10.003, PMID: 18023913

[30] Yang CC. Measuring health indicators and allocating health resources: a DEA-based approach. Health Care Manag Sci. (2017) 20:365–78. doi: 10.1007/s10729-016-9358-2, PMID: 26842823

[31] Li Q Wei J Jiang F Zhou G Jiang R Chen M. Equity and efficiency of health care resource allocation in Jiangsu Province. China Int J Equity Health. (2020) 19:211. doi: 10.1186/s12939-020-01320-2, PMID: 33246458 PMC7694921

[32] Yi M Peng J Zhang L Zhang Y. Is the allocation of medical and health resources effective? Characteristic facts from regional heterogeneity in China. Int J Equity Health. (2020) 19:89. doi: 10.1186/s12939-020-01201-8, PMID: 32513283 PMC7477901

[33] Dong E Liu S Chen M Wang H Chen LW Xu T. Differences in regional distribution and inequality in health-resource allocation at hospital and primary health Centre levels: a longitudinal study in Shanghai. China BMJ Open. (2020) 10:e035635. doi: 10.1136/bmjopen-2019-035635, PMID: 32690509 PMC7371131

[34] Zhikun C Yan Z. Fiscal decentralization and efficiency of medical and health expenditure: as the example of Jiangsu Province. Finance Trade Res. (2018) 29:76–84. doi: 10.19337/j.cnki.34-1093/f.2018.09.007

[35] Su W Du L Fan Y Wang P. Equity and efficiency of public hospitals’ health resource allocation in Guangdong Province, China. Int J Equity Health. (2022) 21:138. doi: 10.1186/s12939-022-01741-1, PMID: 36138478 PMC9493174

[36] Yixin B Hang Z Mingying G Hao W Chuan P. Study on the current situation and equity of health resource allocation in Chengdu-Chongqing economic circle. Chin Hospitals. (2023) 27:14–7. doi: 10.19660/j.issn.1671-0592.2023.04.04

[37] Huang M Luo D Wang Z Cao Y Wang H Bi F. Equity and efficiency of maternal and child health resources allocation in Hunan Province. China BMC Health Serv Res. (2020) 20:300. doi: 10.1186/s12913-020-05185-7, PMID: 32293425 PMC7158093

[38] Yixin B Mingying G Chuan P. Distribution characteristics and prediction of health resources in Chengdu-Chongqing economic circle. Chin Rural Health Serv Admin. (2023) 43:650–6. doi: 10.19955/j.cnki.1005-5916.2023.09.008

[39] Xu R Mu T Liu Y Ye Y Xu C. Trends in the disparities and equity of the distribution of traditional Chinese medicine health resources in China from 2010 to 2020. PLoS One. (2022) 17:e0275712. doi: 10.1371/journal.pone.0275712, PMID: 36215249 PMC9550081

[40] Yaqing L Haoran N Xiangyang T Meicheng Z Feng J Yutong Q. Research on equity of medical resource allocation in Yangtze River Economic Belt under healthy China strategy. Front Public Health. (2023) 11:1175276. doi: 10.3389/fpubh.2023.1175276, PMID: 37435525 PMC10332165

[41] Zhaofei Z Lei H Qi C Bo G Shujuan Y Xu L. Comprehensive evaluation on the level of primary medical and health services, Sichuan. Modern Preventive Medicine. (2021) 17:3161.

[42] Gui J Xiangzheng D Xiaodong Z Baishu G Jun Y. Spatiotemporal patterns in urbanization efficiency within the Yangtze River Economic Belt between 2005 and 2014. J Geogr Sci. (2018) 28:1113–26. doi: 10.1007/s11442-018-1545-2

[43] De P Dhar A Bhattacharya BN. Efficiency of health care system in India: an inter-state analysis using DEA approach. Soc Work Public Health. (2012) 27:482–506. doi: 10.1080/19371918.2012.672261, PMID: 22873937

[44] Zheng Y Xiang Z. Studying on the efficiency measurement of medical resources in the Yellow River Economic Belt under the 14th five-year plan based on DEA and SFA. Chin Health Serv Manag. (2022) 39:81–4.

[45] Zhao N Chen K. Equity and efficiency of medical and health service system in China. BMC Health Serv Res. (2023) 23:33. doi: 10.1186/s12913-023-09025-2, PMID: 36641525 PMC9840836

[46] Seddighi H Nejad FN Basakha M. Health systems efficiency in eastern Mediterranean districts: a data envelopment analysis. Cost Eff Resour Alloc. (2020) 18:22. doi: 10.1186/s12962-020-00217-9, PMID: 32684852 PMC7358927

[47] Dexian B Hongyan L. Efficiency of health resource allocation in the Yangtze River Delta: a data envelopment analysis.Chinese medical. Manag Sci. (2023) 13:32–9.

[48] Xue Y Chengjue W Yu T Nan Z. Spillover effects of online paid medical treatment services on improving offline medical treatment services. Manag Sci. (2023) 3:66–80. doi: 10.3969/j.issn.1672-0334.2023.03.005

[49] Goh JM Gao GG Agarwal R. The creation of social value: can an online health community reduce rural-urban health disparities? MIS Q. (2016) 40:247–63. doi: 10.25300/MISQ/2016/40.1.11

[50] Shi J Yuan R Yan X Wang M Qiu J Ji X. Factors influencing the sharing of personal health data based on the integrated theory of privacy calculus and theory of planned behaviors framework: results of a cross-sectional study of Chinese patients in the Yangtze River Delta. J Med Internet Res. (2023) 25:e46562. doi: 10.2196/46562, PMID: 37410526 PMC10359915

[51] Zhang X Zhao L Cui Z Wang Y. Study on equity and efficiency of health resources and services based on key indicators in China. PLoS One. (2015) 10:e0144809. doi: 10.1371/journal.pone.0144809, PMID: 26679187 PMC4683010

