# Supplementary material for: Equity and efficiency of health resource allocation in the Chengdu–Chongqing Economic Circle of China
Source: Front Public Health. 2024 Aug 27;12:1369568. doi: 10.3389/fpubh.2024.1369568 (PMC11384592; doi:10.3389/fpubh.2024.1369568)
Supplement: Supplementary file 1 [file Table_1.DOCX]

Supplementary Material

**Appendix Table 1** TE of health resource allocation in various districts of Chengdu-Chongqing Economic Circle at stage 1 and stage 3.

| **Districts** | **The first stage** | | | |  | **The third stage** | | | |
| --- | --- | --- | --- | --- | --- | --- | --- | --- | --- |
|  | **2009** | **2011-2015** | **2016-2020** | **2021** |  | **2009** | **2011-2015** | **2016-2020** | **2021** |
| **Chengdu Economic Circle** | 0.454 | 0.832 | 0.868 | 0.863 |  | 0.911 | 0.963 | 0.969 | 0.942 |
| Chengdu | 0.554 | 0.921 | 0.924 | 0.930 |  | 1.000 | 1.000 | 1.000 | 1.000 |
| Zigong | 0.404 | 0.761 | 0.876 | 0.953 |  | 0.797 | 0.954 | 0.983 | 0.980 |
| Mianyang | 0.539 | 0.887 | 0.987 | 0.989 |  | 0.994 | 0.998 | 1.000 | 1.000 |
| Suining | 0.455 | 0.891 | 0.937 | 0.930 |  | 0.860 | 0.927 | 0.961 | 0.952 |
| Luzhou | 0.442 | 0.797 | 0.803 | 0.793 |  | 0.961 | 0.994 | 0.988 | 0.948 |
| Deyang | 0.478 | 0.984 | 1.000 | 1.000 |  | 0.980 | 1.000 | 1.000 | 1.000 |
| Nanchong | 0.544 | 0.866 | 0.881 | 0.860 |  | 1.000 | 0.991 | 0.980 | 0.946 |
| Meishan | 0.334 | 0.853 | 0.852 | 0.907 |  | 0.794 | 1.000 | 0.971 | 1.000 |
| Neijiang | 0.392 | 0.747 | 0.766 | 0.716 |  | 0.880 | 0.972 | 0.945 | 0.854 |
| Leshan | 0.354 | 0.682 | 0.750 | 0.757 |  | 0.825 | 0.871 | 0.919 | 0.882 |
| Yibin | 0.507 | 0.795 | 0.796 | 0.789 |  | 0.977 | 0.988 | 0.991 | 0.938 |
| Guang'an | 0.528 | 0.878 | 0.894 | 0.898 |  | 0.912 | 0.981 | 0.975 | 0.943 |
| Dazhou | 0.430 | 0.769 | 0.820 | 0.723 |  | 0.891 | 0.984 | 1.000 | 0.937 |
| Ya'an | 0.443 | 0.644 | 0.738 | 0.706 |  | 0.886 | 0.790 | 0.817 | 0.748 |
| Ziyang | 0.412 | 1.000 | 1.000 | 1.000 |  | 0.914 | 1.000 | 1.000 | 1.000 |
| **Chongqing Economic Circle** | 0.821 | 0.839 | 0.860 | 0.912 |  | 0.841 | 0.676 | 0.764 | 0.803 |
| Fuling | 0.582 | 0.691 | 0.858 | 0.868 |  | 0.877 | 0.686 | 0.826 | 0.844 |
| Yuzhong | 1.000 | 1.000 | 1.000 | 1.000 |  | 1.000 | 1.000 | 1.000 | 1.000 |
| Shapingba | 0.601 | 0.832 | 0.683 | 0.711 |  | 0.849 | 0.572 | 0.665 | 0.739 |
| Jiulongpo | 0.553 | 0.721 | 0.662 | 0.773 |  | 0.832 | 0.690 | 0.747 | 0.786 |
| Dadukou | 0.671 | 0.635 | 0.715 | 0.810 |  | 0.442 | 0.305 | 0.421 | 0.485 |
| Jiangbei | 0.541 | 0.824 | 0.839 | 0.867 |  | 0.823 | 0.569 | 0.734 | 0.771 |
| Changshou | 0.633 | 0.650 | 0.722 | 0.784 |  | 0.685 | 0.551 | 0.636 | 0.615 |
| Jiangjin | 1.000 | 0.802 | 0.925 | 0.825 |  | 1.000 | 0.825 | 0.918 | 0.857 |
| Nan'an | 0.559 | 0.778 | 0.777 | 0.967 |  | 0.695 | 0.490 | 0.626 | 0.738 |
| Beibei | 0.613 | 0.728 | 0.800 | 0.925 |  | 0.788 | 0.561 | 0.621 | 0.692 |
| Yubei | 0.560 | 0.729 | 0.773 | 0.997 |  | 0.740 | 0.664 | 0.779 | 0.902 |
| Ba'nan | 0.723 | 0.804 | 0.793 | 0.929 |  | 0.873 | 0.766 | 0.816 | 0.842 |
| Hechuan | 1.000 | 0.783 | 0.732 | 0.745 |  | 1.000 | 0.640 | 0.736 | 0.732 |
| Yongchuan | 1.000 | 0.958 | 0.944 | 1.000 |  | 1.000 | 0.875 | 0.922 | 1.000 |
| Nanchuan | 1.000 | 0.914 | 0.841 | 0.914 |  | 0.774 | 0.604 | 0.696 | 0.734 |
| Qijiang | 0.824 | 0.765 | 0.799 | 0.755 |  | 0.878 | 0.777 | 0.804 | 0.784 |
| Dazu | 1.000 | 1.000 | 0.986 | 0.888 |  | 0.997 | 0.815 | 0.895 | 0.796 |
| Bishan | 1.000 | 0.819 | 0.810 | 1.000 |  | 0.765 | 0.544 | 0.666 | 0.758 |
| Tongliang | 0.767 | 0.853 | 0.940 | 1.000 |  | 0.815 | 0.566 | 0.688 | 0.874 |
| Tongnan | 0.947 | 1.000 | 1.000 | 1.000 |  | 0.843 | 0.741 | 0.813 | 0.751 |
| Rongchang | 0.851 | 0.954 | 0.929 | 1.000 |  | 0.854 | 0.630 | 0.752 | 0.777 |
| Wanzhou | 0.770 | 0.886 | 0.987 | 0.913 |  | 1.000 | 0.850 | 0.939 | 0.910 |
| Qianjiang | 1.000 | 1.000 | 1.000 | 1.000 |  | 0.846 | 0.633 | 0.784 | 0.859 |
| Kaizhou | 0.941 | 0.891 | 0.945 | 1.000 |  | 0.944 | 0.779 | 0.910 | 1.000 |
| Liangping | 0.745 | 0.745 | 0.808 | 0.848 |  | 0.678 | 0.633 | 0.674 | 0.674 |
| Fengdu | 1.000 | 0.909 | 0.897 | 0.979 |  | 0.790 | 0.674 | 0.683 | 0.725 |
| Dianjiang | 1.000 | 1.000 | 0.972 | 1.000 |  | 0.991 | 0.761 | 0.807 | 0.909 |
| Zhongxian | 0.927 | 0.742 | 0.839 | 0.959 |  | 0.674 | 0.644 | 0.723 | 0.798 |
| Yunyang | 1.000 | 0.905 | 0.972 | 1.000 |  | 0.942 | 0.745 | 0.878 | 0.923 |
| **Overall** | 0.696 | 0.836 | 0.863 | 0.896 |  | 0.865 | 0.774 | 0.834 | 0.850 |

Note: 2011-2015 is the average TE during “the 12th Five-Year Plan” period, and 2016-2020 is the average TE during “the 13th Five-Year Plan” period.

**Appendix Table 2** MPI and its decomposition in each district of Chengdu-Chongqing Economic Circle from 2009 to 2021.

| **Districts** | **Effch** | **Techch** | **Pech** | **Sech** | **Tfpch** |
| --- | --- | --- | --- | --- | --- |
| **Chengdu Economic Circle** | 1.003 | 1.033 | 1.005 | 0.998 | 1.036 |
| Chengdu | 1.000 | 1.035 | 1.000 | 1.000 | 1.035 |
| Zigong | 1.017 | 1.037 | 1.019 | 0.999 | 1.055 |
| Mianyang | 1.001 | 1.052 | 1.000 | 1.001 | 1.053 |
| Suining | 1.009 | 1.014 | 1.011 | 0.998 | 1.023 |
| Luzhou | 0.999 | 1.043 | 1.003 | 0.996 | 1.042 |
| Deyang | 1.002 | 1.027 | 1.002 | 1.000 | 1.028 |
| Nanchong | 0.995 | 1.015 | 1.000 | 0.995 | 1.011 |
| Meishan | 1.019 | 1.046 | 1.019 | 1.000 | 1.067 |
| Neijiang | 0.998 | 1.027 | 1.003 | 0.995 | 1.024 |
| Leshan | 1.006 | 1.034 | 1.008 | 0.997 | 1.040 |
| Yibin | 0.997 | 1.051 | 1.001 | 0.996 | 1.047 |
| Guang'an | 1.003 | 1.025 | 0.999 | 1.003 | 1.028 |
| Dazhou | 1.004 | 1.032 | 1.009 | 0.996 | 1.036 |
| Ya'an | 0.986 | 1.033 | 0.996 | 0.990 | 1.018 |
| Ziyang | 1.008 | 1.028 | 1.007 | 1.000 | 1.036 |
| **Chongqing Economic Circle** | 0.996 | 1.026 | 1.000 | 0.997 | 1.023 |
| Fuling | 0.997 | 1.017 | 1.000 | 0.997 | 1.014 |
| Yuzhong | 1.000 | 1.064 | 1.000 | 1.000 | 1.064 |
| Shapingba | 0.988 | 1.051 | 0.991 | 0.997 | 1.039 |
| Jiulongpo | 0.995 | 1.055 | 0.999 | 0.996 | 1.050 |
| Dadukou | 1.008 | 1.029 | 1.000 | 1.007 | 1.038 |
| Jiangbei | 0.995 | 1.052 | 1.005 | 0.990 | 1.046 |
| Changshou | 0.991 | 1.013 | 1.000 | 0.991 | 1.004 |
| Jiangjin | 0.987 | 0.996 | 0.997 | 0.990 | 0.983 |
| Nan'an | 1.005 | 1.039 | 1.006 | 0.999 | 1.044 |
| Beibei | 0.989 | 1.039 | 1.003 | 0.986 | 1.028 |
| Yubei | 1.017 | 1.046 | 1.003 | 1.013 | 1.063 |
| Ba'nan | 0.997 | 1.025 | 1.003 | 0.994 | 1.022 |
| Hechuan | 0.974 | 1.016 | 0.992 | 0.983 | 0.990 |
| Yongchuan | 1.000 | 1.025 | 1.000 | 1.000 | 1.025 |
| Nanchuan | 0.996 | 1.028 | 1.000 | 0.996 | 1.023 |
| Qijiang | 0.991 | 1.035 | 0.995 | 0.995 | 1.025 |
| Dazu | 0.981 | 1.023 | 0.999 | 0.983 | 1.004 |
| Bishan | 0.999 | 1.022 | 1.000 | 0.999 | 1.022 |
| Tongliang | 1.006 | 1.041 | 1.002 | 1.004 | 1.047 |
| Tongnan | 0.990 | 0.995 | 1.000 | 0.990 | 0.985 |
| Rongchang | 0.992 | 1.010 | 1.001 | 0.991 | 1.002 |
| Wanzhou | 0.992 | 1.016 | 0.999 | 0.994 | 1.008 |
| Qianjiang | 1.001 | 1.069 | 1.000 | 1.001 | 1.071 |
| Kaizhou | 1.005 | 1.027 | 1.000 | 1.005 | 1.032 |
| Liangping | 0.999 | 1.010 | 1.000 | 1.000 | 1.009 |
| Fengdu | 0.993 | 1.007 | 1.000 | 0.993 | 1.000 |
| Dianjiang | 0.993 | 1.017 | 1.000 | 0.993 | 1.010 |
| Zhongxian | 1.014 | 0.988 | 1.000 | 1.014 | 1.002 |
| Yunyang | 0.998 | 1.006 | 1.000 | 0.998 | 1.004 |

**
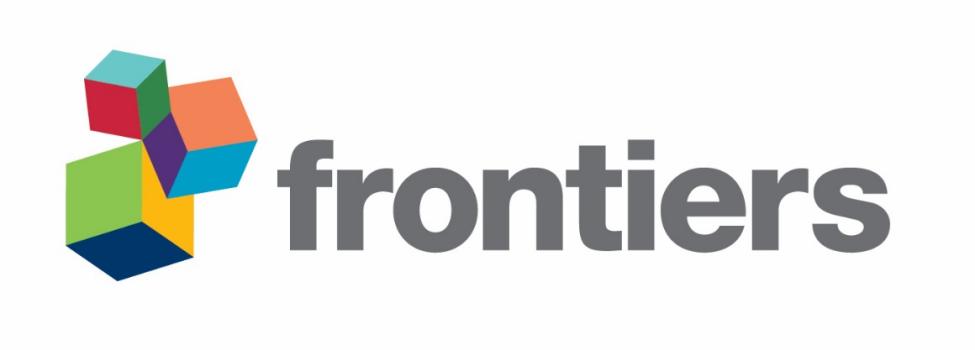
**
